# *Streptococcus pneumoniae* Serotypes Associated with Death, South Africa, 2012–2018

**DOI:** 10.3201/eid2801.210956

**Published:** 2022-01

**Authors:** Annelies Müller, Jackie Kleynhans, Linda de Gouveia, Susan Meiring, Cheryl Cohen, Lucy Jane Hathaway, Anne von Gottberg, for GERMS-SA

**Affiliations:** University of Bern Graduate School for Cellular and Biomedical Sciences, Bern, Switzerland (A. Müller);; University of Bern Institute for Infectious Diseases, Bern (A. Müller, L.J. Hathaway);; National Institute for Communicable Diseases of the National Health Laboratory Service, Johannesburg, South Africa (J. Kleynhans, L. de Gouveia, S. Meiring, C. Cohen, A. von Gottberg);; University of the Witwatersrand, Johannesburg (J. Kleynhans, S. Meiring, C. Cohen, A. von Gottberg); 1 These authors contributed equally to this article.

**Keywords:** *Streptococcus pneumoniae*, streptococci, *Streptococcus*, bacteria, meningitis/encephalitis, serotype, mortality, polysaccharide capsule, South Africa, case-fatality ratio, Switzerland

## Abstract

The *Streptococcus pneumoniae* polysaccharide capsule plays a role in disease severity. We assessed the association of serotype with case-fatality ratio (CFR) in invasive pneumococcal disease (IPD) and meningitis in South Africa, 2012–2018 (vaccine era), using multivariable logistic regression by manual backward elimination. The most common serotypes causing IPD were 8 and 19A. In patients <15 years of age, serotypes associated with increased CFR in IPD, compared with serotype 8 and controlling for confounding factors, were 11A, 13, 19F, 15A, and 6A. None of these serotypes were associated with increased CFR in meningitis. Among IPD patients >15 years of age, serotype 15B/C was associated with increased CFR. Among meningitis patients of all ages, serotype 1 was associated with increased CFR. PCV13 serotypes 1, 3, 6A, 19A, and 19F should be monitored, and serotypes 8, 12F, 15A, and 15B/C should be considered for inclusion in vaccines to reduce deaths caused by *S. pneumoniae*.

*Streptococcus pneumoniae* is a leading cause of bacterial pneumonia and invasive pneumococcal disease (IPD), including meningitis, worldwide. These diseases especially affect the young, the elderly, and the immunocompromised (*1*). The pneumococcal capsular polysaccharide is a primary virulence factor used to classify the bacteria into >100 different serotypes; it is the basis of pneumococcal conjugate vaccines (PCVs).

Continued surveillance and choice of serotypes to include in future vaccination remains key to effective prevention strategies. PCV13, containing 13 different serotypes (1, 3, 4, 5, 6A, 6B, 7F, 9V, 14, 19A, 19F, 18C, and 23F), is the most commonly used pneumococcal vaccine; clinical trials are ongoing for new vaccines (*2*–*5*). Choosing which serotypes to include in vaccination strategies is important because serotype prevalence varies geographically, introduction of PCVs has changed serotype prevalence (*6*–*8*), and some serotypes are more common or are associated with more severe disease than other serotypes. In South Africa, before PCVs were introduced, the most commonly occurring serotypes in adults and adolescents >15 years of age were 1, 19A, 4, and 3; serotypes 1 and 19F were associated with death (*9*). The association between serotype and disease outcome has also been shown in other epidemiologic studies (*10*–*14*) as well as in vivo models (*15*,*16*). Serotypes repeatedly associated with elevated case-fatality ratio (CFR) in various countries are 3, 6B, 9N, 11A, 16F, 19F, and 19A in adults (*14*) and 19F, 6A, and 3 in children (*17*). South Africa introduced the 7-valent PCV (PCV7) into the country’s expanded program on immunization in 2009. In 2011, PCV7 was replaced by PCV13 (*10*). The current national vaccine program in South Africa recommends 3 doses of the PCV13 vaccine at 6 weeks, 14 weeks, and 9 months of age.

In this study, we aimed to determine which serotypes are associated with increased CFR in IPD and meningitis patients in the vaccine era in South Africa. We conducted the analysis for all IPD and for a subset of meningitis patients and in all ages as well as in 2 age groups of <15 and >15 years. Our aims were to identify possible differences of serotypes associated with death in children and adults and between total IPD and meningitis and to compare the results with published data from the prevaccine era.

The Human Research Ethics Committee (Medical) of University of Witwatersrand (Johannesburg, South Africa) and relevant university and provincial ethics committees approved the GERMS-SA surveillance study (clearance nos. M140159, M081117, M021042, M180101). The Kantonale Ethikkommission Bern approved analysis of encoded South African patient data in Switzerland (project ID 2018–01172).

## Methods

### IPD Surveillance 

We conducted IPD surveillance through GERMS-SA (https://www.nicd.ac.za/germs), an active laboratory-based surveillance program in South Africa for which information on bacterial and fungal pathogens including *S. pneumoniae* is collected. Specimens with laboratory-confirmed IPD from both the public and private sector are sent to the National Institute for Communicable Diseases (NICD) in Johannesburg as part of the GERMS-SA program. Demographic information is collected for all GERMS-SA cases. For 28 enhanced surveillance sites across South Africa, additional clinical information is collected on IPD patients, including outcome and HIV status, which we used for this study.

We performed pneumococcal serotyping by Quellung reaction using specific antiserum (Statens Serum Institute, https://en.ssi.dk). We determined serotype of nonviable isolates by PCR where possible. Serotype 15B and 15C are reported as 15B/C because of reported reversible switching between them (*18*).

### Study Population and Case Definition

**Figure 1:**
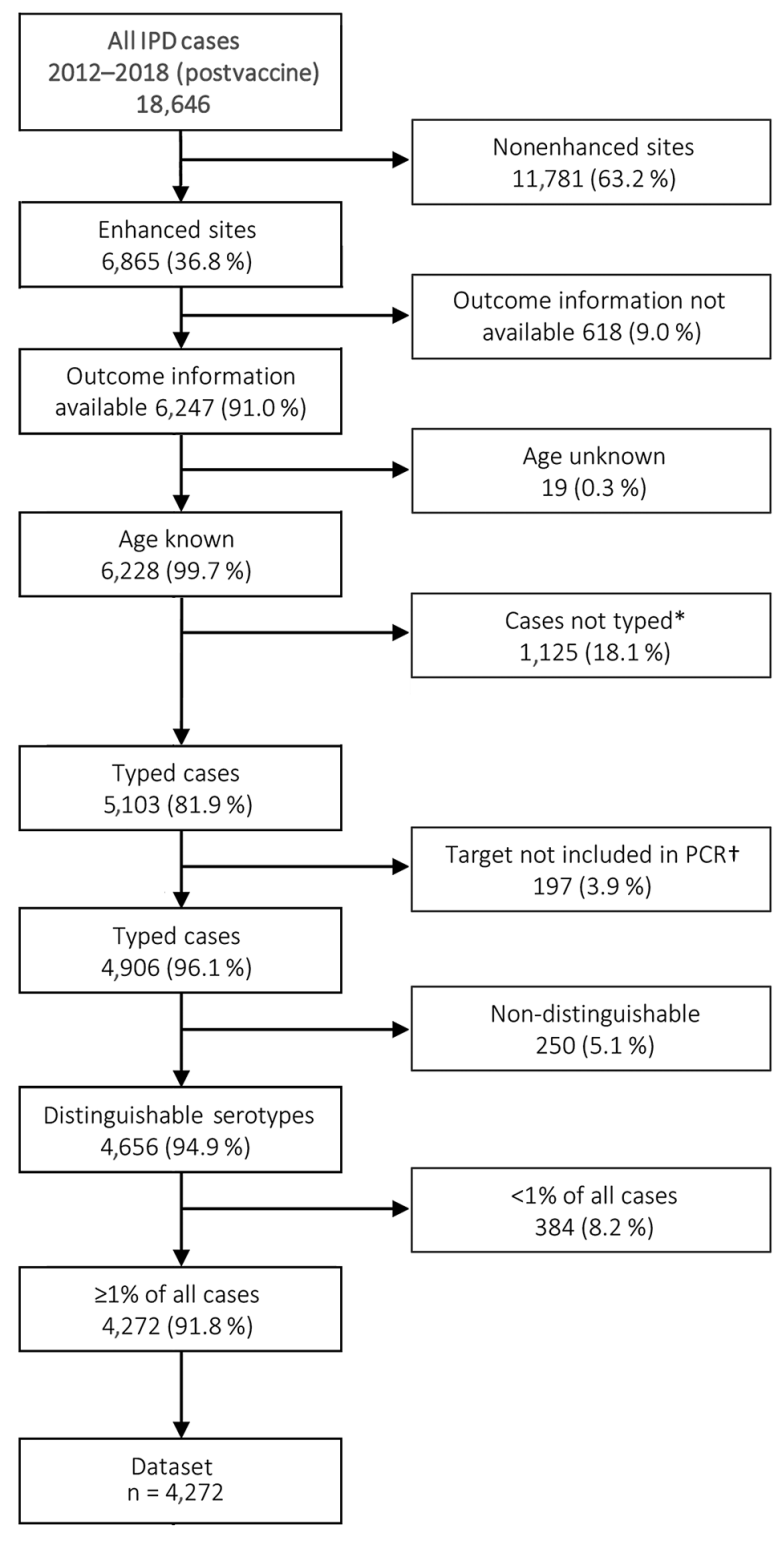
Flow diagram of cases included in the analysis of patients with IPD in South Africa, 2012–2018. Asterisk (*) indicates that cases were not typed for 1 of the following reasons: the case was identified by the laboratory information system data audits, n = 651; the sample was positive for pneumococcal antigen detection but nonviable upon culture, n = 14; the isolate was not received and thus serotyping not possible, n = 379; or sample was nonviable and PCR negative, n = 81. Dagger (†) indicates that samples for which Quellung did not yield a valid result were prepared for PCR confirmation and serotyping if PCR positive. If the multiplex PCR was negative for all 38 detectable serotypes, then the sample was excluded from analysis. IPD, invasive pneumococcal disease.

We included all IPD cases from enhanced surveillance sites from the GERMS-SA program during January 1, 2012–December 31, 2018, for which age, in-hospital outcome, and serotype information were available (Figure 1). We categorized 7 age groups: <1 year, 1–4 years, 5–14 years, 15–24 years, 25–44 years, 45–64 years, and >64 years. Of all IPD cases with serotype information available, we excluded cases if the patient was infected with >1 serotype or if the serotype was not in the currently available pneumococcal conjugate vaccines (PCV) or the 23-valent polysaccharide vaccine (PPSV23) and made up <1% of isolates from cases. We also excluded cases if the serotype could not be distinguished because initial rapid antigen test confirming the presence of pneumococcal antigen was performed at initial laboratory but culture was negative and a PCR assay to identify serotype was negative or the serotype was not distinguishable within a group of serotypes. IPD was defined if *S. pneumoniae* was cultured from a patient sample of a normally sterile site (e.g., cerebrospinal fluid [CSF], blood, joint, or pleural fluid) or if the sample tested positive for *S. pneumoniae* by PCR. We defined a case as a meningitis case if the attending doctor diagnosed meningitis, regardless of the specimen (CSF, blood, or other) that was taken. We excluded repeat isolates from the same patient within 21 days of positive result. We used in-hospital death within 30 days of IPD episode as the measure for death outcome.

### Statistical Analysis

We used a multivariable logistic regression by manual backward elimination. In a first step, we assessed the effect of age, HIV status, sex, province, race, year, diagnosis, and underlying medical condition using a univariate model. We included all significant variables (p<0.2) in the multivariable logistic regression. In the multivariable model, we dropped nonsignificant factors (p>0.05) with manual backward elimination. We conducted statistical analysis using Stata version 16.0 (StataCorp, https://www.stata.com). We performed all statistical tests with all IPD cases. We analyzed nonmeningitis IPD cases (nmIPD) as a supplementary analysis. We used only meningitis cases in our primary analysis because meningitis has a distinct clinical manifestation; the separate analysis enabled us to learn if the serotypes associated with CFR in all IPD cases are also those present in meningitis cases. In addition, we performed all analysis with all ages and also with age groups <15 years and >15 years because these age groups are often described in literature; we separated by age group for easier comparison to previous studies. We considered results in the final model significant for p<0.01 after Bonferroni correction. To compare CFR between serotypes, we chose serotype 8 as the reference because it was the most commonly occurring serotype in all IPD cases (507/4,272, 11.9%). Each of the other serotypes accounted for <10% of total isolates.

## Results

**Figure 2:**
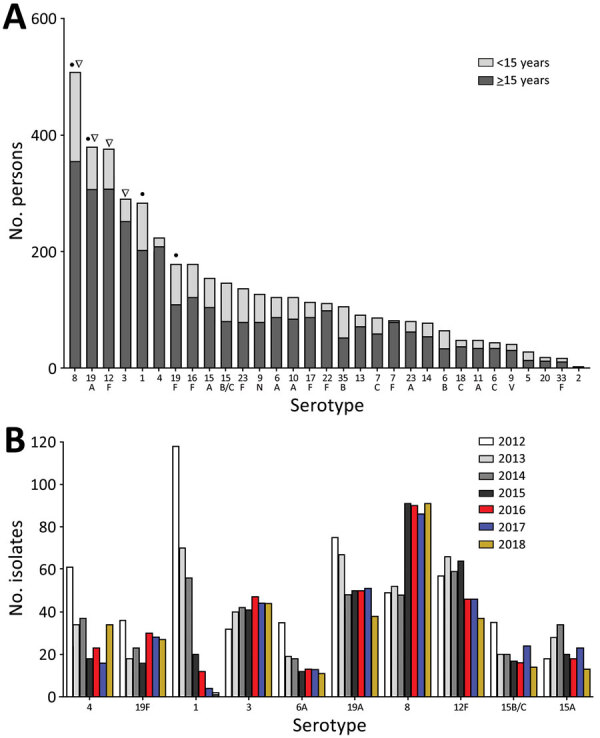
Number of isolates per serotype in invasive pneumococcal disease patients in South Africa, 2012–2018. A) Total number of isolates; serotype 8 was the most commonly isolated (507, 12%). Black dots indicate most common serotypes in patients <15 years of age; arrowheads indicate most common serotypes in patients >15 years of age. B) Number of isolates per serotype per year of the 4 most common serotypes in the prevaccine era (1, 19A, 3, and 4) (*9*), the 4 most common in the vaccine era (8, 19A, 12F, and 3), and 6A and 19F. The 7-valent pneumococcal conjugate vaccine was introduced in 2009, 13-valent in 2011.

A total of 18,646 IPD patients were reported to the GERMS-SA surveillance program during 2012–2018 (Figure 1). Of these, 6,865 (36.8%) patients were enrolled in the GERMS-SA enhanced surveillance program and 6,247 (91.0%) of them had available outcome information. Of patients with outcome information, 6,228 (99.97%) had age information. Samples from 5,103 (81.9%) with known age were serotyped; of those, 4,906 (96.1%) yielded a valid serotype by Quellung or were positive for one of the 38 serotypes included in the multiplex PCR. Of those with valid serotypes, 4,656 (94.9%) had distinguishable serotype data. Of the distinguishable serotype cases, 4,272 (91.8%) cases had serotypes included in the PCV-13 vaccine or serotypes that comprised >1% of all cases. The cases used in this study made up 22.9% of overall IPD cases (4,272/18,646). The most represented age group was 25–44 years (40.7%) (Table 1), which was similar to that of all cases, for which the most represented age group was also 25–44 years (Appendix 2 Table 1). Of those with known sex, 51.2% were female, was similar to the distribution of sex of all cases (51.2% female of those with known sex) (Appendix 2 Table 1). Of those with known HIV status, 67.0% were HIV positive (Table 2; Appendix 1 Figure 2).

**Table 1:** Demographic characteristics of patients with invasive pneumococcal disease, South Africa, 2012–2018*

Variable	No. (%) patients		
	Total IPD cases, N = 4,272	Total <15 y, n = 1,095	Total >15 y, n = 3,177
Year of specimen collection			
2012	792 (18.5)	237 (21.6)	555 (17.5)
2013	679 (15.99)	193 (17.6)	486 (15.3)
2014	601 (14.1)	194 (17.7)	407 (12.8)
2015	603 (14.1)	138 (12.6)	465 (14.6)
2016	585 (13.7)	136 (12.4)	449 (14.1)
2017	553 (12.9)	106 (9.7)	447 (14.1)
2018	459 (10.7)	91 (8.3)	368 (11.6)
Province			
Eastern Cape	272 (6.4)	55 (5.0)	217 (6.8)
Free State	221 (5.2)	83 (7.6)	138 (4.3)
Gauteng	1,636 (38.3)	417 (38.1)	1,219 (38.4)
KwaZulu-Natal	685 (16.0)	180 (16.4)	505 (15.9)
Limpopo	76 (1.8)	20 (1.9)	56 (1.8)
Mpumalanga	172 (4.0)	33 (3.0)	139 (4.4)
Northern Cape	214 (5.0)	36 (3.3)	178 (5.6)
North West	119 (2.8)	26 (2.4)	93 (2.9)
Western Cape	877 (20.5)	245 (22.4)	632 (19.9)
Sex			
F	2,184/4,267 (51.2)	505/1,093 (46.2)	1,679/3,174 (52.9)
M	2,083/4,267 (48.8)	588/1,093 (53.8)	1,495/3,174 (47.1)
Unknown	5/4,272 (0.1)	2/1,095 (0.2)	3/1,095 (0.1)
Positive HIV status	2,325/3,468 (67.0)	318/905 (35.1)	2,007/2,563 (78.3)
Age group			
<1 y	504 (11.8)	504 (46.0)	NA
1–4 y	319 (7.5)	319 (29.1)	NA
5–14 y	272 (6.4)	272 (24.8)	NA
15–24 y	282 (6.6)	NA	282 (8.9)
25–44 y	1,738 (40.7)	NA	1738 (54.7)
45–64 y	917 (21.5)	NA	917 (28.9)
>65 y	240 (5.6)	NA	240 (7.6)
Race			
Black	3,452/4,160 (83.0)	939/1,066 (88.1)	2,513/3,094 (81.2)
All others	708/4,160 (17.0)	127/1,066 (11.9)	581/3,094 (18.8)

*Data from GERMS-SA (https://www.nicd.ac.za/germs) enhanced surveillance sites. NA, not applicable.

**Table 2:** Clinical characteristics of patients with invasive pneumococcal disease, South Africa, 2012–2018*

Variable	No. (%) patients		
	Total IPD cases, N = 4,272	Total <15 y, n = 1,095	Total >15 y, n = 3,177
Serotype†			
4	223 (5.2)	15 (1.4)	208 (6.6)
6B	64 (1.5)	30 (2.7)	34 (1.1)
9V	41 (1.0)	10 (0.9)	31 (1.0)
14	77 (1.8)	23 (2.1)	54 (1.7)
18C	48 (1.1)	11 (1.0)	37 (1.2)
19F	178 (4.2)	69 (6.3)	109 (3.4)
23F	136 (3.2)	57 (5.2)	79 (2.5)
1	282 (6.6)	80 (7.3)	202 (6.4)
3	290 (6.8)	39 (3.6)	251 (7.9)
5	28 (0.7)	14 (1.3)	14 (0.4)
6A	121 (2.8)	34 (3.1)	87 (2.8)
7F	81 (1.9)	2 (0.2)	79 (2.5)
19A	379 (8.9)	73 (6.7)	306 (9.6)
2	2 (0.1)	0	2 (0.1)
8	507 (11.9)	153 (14.0)	354 (11.1)
9N	127 (3.0)	15 (1.4)	112 (3.5)
10A	121 (2.8)	37 (3.4)	84 (2.6)
11A	48 (1.1)	14 (1.3)	34 (1.1)
12F	375 (8.8)	68 (6.2)	307 (9.7)
15B/C	146 (3.4)	66 (6.0)	80 (2.5)
17F	113 (2.7)	26 (2.4)	87 (2.7)
20	19 (0.4)	6 (0.6)	13 (0.4)
22F	111 (2.6)	12 (1.1)	99 (3.1)
33F	17 (0.4)	6 (0.6)	11 (0.4)
16F	178 (4.2)	57 (5.2)	121 (3.8)
15A	154 (3.6)	50 (4.6)	104 (3.3)
13	91 (2.1)	20 (1.8)	71 (2.2)
7C	86 (2.0)	27 (2.5)	59 (1.7)
35B	105 (2.5)	53 (4.8)	52 (1.6)
23A	80 (1.9)	18 (1.6)	62 (2.0)
6C	44 (1.0)	10 (0.9)	34 (1.1)
Specimen			
CSF	1,223 (28.6)	328 (30.0)	895 (28.2)
Blood	2,728 (63.9)	697 (63.7)	2,031 (63.9)
Other	321 (7.5)	70 (6.4)	251 (7.9)
Diagnosis			
Bacteremia without focus	319 (7.5)	94 (8.6)	225 (7.1)
Lower respiratory tract infection	2,352 (55.1)	551 (50.3)	1,801 (56.7)
Meningitis‡	4,439 (33.7)	400 (36.5)	1,039 (32.7)
Other	162 (3.6)	50 (4.6)	112 (3.5)
In-hospital outcome			
Died	1,369 (32.1)	242 (22.1)	1,127 (35.5)
Underlying conditions§	1,352 (31.7)	428 (39.1)	924 (29.1)

*Data from GERMS-SA (https://www.nicd.ac.za/germs) enhanced surveillance sites. †Serotypes appear in order of vaccine. The first 7 are in the PCV7 vaccine, the first 13 are in the PCV13 vaccine, the first 23 in the PPSV23 vaccine, and the rest are not included in a current vaccine. ‡Meningitis cases include those diagnosed with meningitis and those with both meningitis and lower respiratory tract infections. §Predisposing conditions defined as any one or more of the following: burns, chronic lung disease (including asthma, chronic obstructive pulmonary disorder, cystic fibrosis), chronic liver disease, chronic renal disease, cardiac conditions (including valvular disease and heart failure), stroke, neuromuscular diseases, cerebral palsy, metabolic diseases (including diabetes mellitus), head injury, surgery, cerebrospinal fluid leaks, ventricular shunts, cochlear implants, primary immunodeficiency conditions, complement deficiency, immunosuppression treatment (steroids/chemo/cancer treatment),protein-energy malnutrition, functional or anatomic asplenia (including sickle cell disease), malignancy, organ transplant, chromosomal conditions (including Down syndrome), prematurity and aplastic anemia.

**Figure 3:**
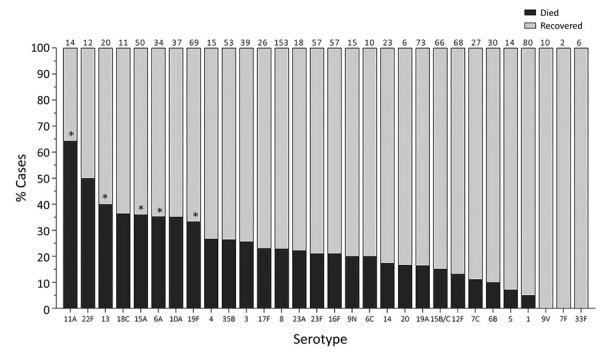
In-hospital outcome per serotype of IPD patients <15 years of age, South Africa, 2012–2018. Numbers above bars indicate number of cases per serotype. Asterisk (*) indicates serotypes significantly associated with increased in-hospital death upon multivariable analysis compared to serotype 8. IPD, invasive pneumococcal disease.

**Figure 4:**
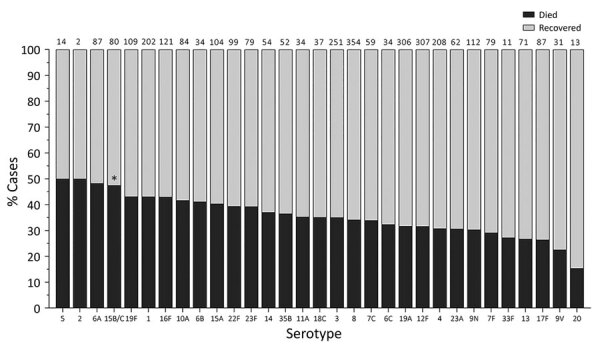
In-hospital outcome per serotype of IPD patients >15 years of age, South Africa, 2012–2018. Numbers above bars indicate number of cases per serotype. Asterisk (*) indicates serotypes significantly associated with increased in-hospital death upon multivariable analysis compared to serotype 8. IPD, invasive pneumococcal disease.

**Figure 5:**
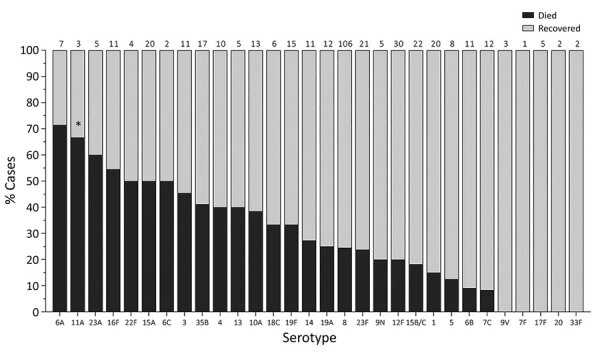
In-hospital outcome per serotype of meningitis patients <15 years of age, South Africa, 2012–2018. A case was defined as a meningitis case if the attending doctor diagnosed it as meningitis, regardless of the specimen type (cerebrospinal fluid, blood, or other) that was taken. Numbers above bars indicate number of cases per serotype. Asterisk (*) indicates serotypes significantly associated with increased in-hospital death upon multivariable analysis compared to serotype 8. IPD, invasive pneumococcal disease.

**Figure 6:**
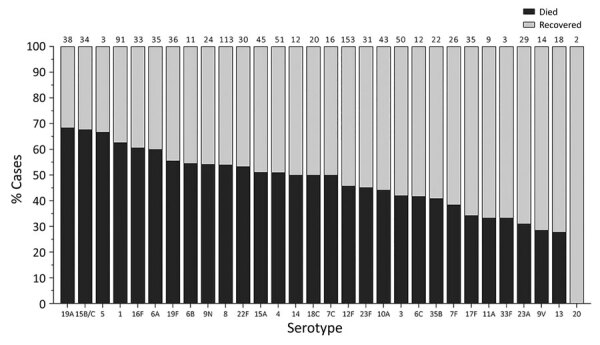
In-hospital outcome per serotype of meningitis patients >15 years of age, South Africa, 2012–2018. A case was defined as a meningitis case if the attending doctor diagnosed it as meningitis, regardless of the specimen type (cerebrospinal fluid, blood, or other) that was taken. Numbers above bars indicate number of cases per serotype. Asterisk (*) indicates serotypes significantly associated with increased in-hospital death upon multivariable analysis compared to serotype 8. IPD, invasive pneumococcal disease.

When we considered all IPD cases and all ages, the most common serotype identified was serotype 8 (11.9%), followed by 19A (8.9%), 12F (8.8%), and 3 (6.8%) (Table 2; Figure 2, panel A). This serotype distribution was similar to that of all 18,646 IPD cases from all GERMS-SA sites, in which the most common serotype identified was also serotype 8 (7.2%), followed by 12F (6.1%), 19A (6.0%), and 3 (4.2%) (Appendix 1 Figure 3; Appendix 2 Table 1). When considering all IPD cases and all ages, we found serotype 1, 6A, 19A, and 12F to decrease in total number of isolates over time period of study (Figure 2, panel B). In patients <15 years of age, the most common serotype was serotype 8 (14.0%), followed by serotype 1 (7.3%), 19A (6.7%), and 19F (6.3%) (Table 2; Figure 2, panel A). Serotypes 12F, 3, 1, and 6A consistently decreased over time of study in this age group (Appendix 1 Figure 4). In patients >15 years of age, the most common serotype was also serotype 8 (11.1%), followed by 12F (9.7%), 19A (9.6%), and 3 (7.9%) (Table 2; Figure 2, panel A). In this age group, we found serotype 1 to consistently decrease over the time period of the study (Appendix 1 Figure 5). The overall CFR was 32.1% when considering all IPD cases. In patients <15 years of age, CFR was 22.1%, and in those >15 years of age, CFR was 35.5% (Tables 1, 2). Serotypes with the highest CFR in all IPD cases were 6A (44.6%), 11A (43.8%), and 22F (40.5%). In IPD patients <15 years, the serotypes with the highest CFR were serotype 11A (64.3%), 22F (50.0%), and 13 (40.0%) (Figure 3). In IPD patients >15 years of age, serotypes with the highest CFR were 5 (50.0%), 6A (48.3%), 15B/C (47.5%), and 19F (43.1%) (Figure 4). In nmIPD patients of all ages, the most common serotype was 19A, followed by 8 and 3 (Appendix 1 Figure 6). The serotype with the highest CFR in nmIPD in all ages was serotype 11A (44.4%), followed by 10A (36.9%) and 6A and 19F (both 35.4%) (Appendix 2 Table 2).

When we analyzed only meningitis cases but considered all ages, we found that serotype 6A (61.9%) had the highest CFR, followed by 16F (59.0%) and 19A (58.0%). Serotype 6A also had the highest CFR when restricted to meningitis patients <15 years of age (71.4%) (Figure 5). In meningitis patients >15 years of age, serotype 1 had the highest CFR (62.6%) (Figure 6).

### Multivariable Analysis of In-Hospital Outcome in IPD

On multivariable analysis, we found that in-hospital death was more likely when patients were infected with serotypes 6A (OR 2.1, 95% CI 1.3–3.4) or 19F (OR 1.9, 95% CI 1.2–2.9) compared with serotype 8. (Appendix 2 Table 3). Other factors significantly associated with in-hospital death on multivariable analysis were positive HIV infection (OR 1.3, 95% CI 1.1–1.6), meningitis (OR 2.6, 95% CI 2.2–3.0), and age. Compared with patients 5–14 years of age, those at greatest risk were <1 year of age (OR 3.3, 95% CI 2.0–5.5), 45–64 years (OR 5.6, 95% CI 3.5–8.9), and those >65 years (OR 8.6, 95% CI 4.8–15.4) (Appendix 2 Table 3). In nmIPD multivariable analysis, serotypes significantly associated with death compared with serotype 8 were 19F, 10A, and 23F (Appendix 2 Table 2).

When restricting analysis to patients <15 years of age, we found that those infected with the following serotypes were associated with increased in-hospital death compared with serotype 8: 11A (OR 11.1, 95% CI 3.3–37.8), 13 (OR 4.3, 95% CI 1.5–12.0), 19F (OR 3.5, 95% CI 1.7–7.1), 6A (OR 3.2, 95% CI 1.4–7.5), and 15A (OR 3.3, 95% CI 1.6–6.9) (Tables 3, 4). Other factors significantly associated with in-hospital death on multivariable analysis were age (<1 year, OR 3.1 [95% CI 1.9–5.1] vs. 5–14 years), meningitis (OR 1.8, 95% CI 1.3–2.6), and province (Table 3). In the nmIPD multivariable analysis of patients <15 years of age, compared with serotype 8, serotype 11A was significantly associated with death (Appendix 2 Table 4).

**Table 3:** Multivariable analysis of factors associated with in-hospital death among patients <15 years of age with invasive pneumococcal disease, South Africa, 2012–2018*

Risk factor	No. deaths/total no. (%)	Unadjusted OR (95% CI)	p value	Adjusted OR (95% CI)	p value
Serotype					
4	4/15 (26.7)	1.2 (0.4–4.1)	0.740	2.2 (0.6–8.1)	0.231
6B	3/30 (10.0)	0.4 (0.1–1.3)	0.124	0.6 (0.2–2.4)	0.492
14	4/23 (17.4)	0.7 (0.2–2.2)	0.556	1.2 (0.4–3.9)	0.777
18C	4/11 (36.4)	1.9 (0,5–7)	0.317	3.1 (0.8–12.2)	0.104
19F	23/69 (33.3)	1.7 (0.9–3.2)	0.102	3.5 (1.7–7.1)	<0.001
23F	12/57 (21.1)	0.9 (0.4–1.9)	0.778	1.9 (0.9–4.4)	0.108
1	4/80 (5.0)	0.2 (0.1–0.5)	0.002	0.6 (0.2–1.8)	0.328
3	10/39 (25.6)	1.2 (0.5–2.6)	0.716	2.1 (0.8–5)	0.115
5	1/14 (7.1)	0.3 (0–2.1)	0.201	0.3 (0–2.1)	0.200
6A	12/34 (35.3)	1.8 (0.8–4.1)	0.135	3.2 (1.4–7.5)	0.008
19A	12/73 (16.4)	0.7 (0.3–1.4)	0.267	1.2 (0.5–2.6)	0.646
8	35/153 (22.9)	Referent	Referent	Referent	Referent
9N	3/15 (20.0)	0.8 (0.2–3.2)	0.800	1.5 (0.4–6)	0.583
10A	13/37 (35.1)	1.8 (0.8–4)	0.127	2.8 (1.2–6.5)	0.015
11A	9/14 (64.3)	6.1 (1.9–19.3)	0.002	11.1 (3.3–37.8)	<0.001
12F	9/68 (13.2)	0.5 (0.2–1.1)	0.102	0.8 (0.3–1.8)	0.545
15B/C	10/66 (15.2)	0.6 (0.3–1.3)	0.197	1 (0.5–2.3)	0.941
17F	6/26 (23.1)	1 (0.4–2.7)	0.982	1.8 (0.6–5.2)	0.260
20	1/6 (16.7)	0.7 (0.1–6)	0.723	0.9 (0.1–8.8)	0.940
22F	6/12 (50.0)	3.4 (1–11.1)	0.046	5 (1.4–18.1)	0.015
16F	12/57 (21.1)	0.9 (0.4–1.9)	0.778	1.5 (0.7–3.3)	0.317
15A	18/50 (36.0)	1.9 (1–3.8)	0.069	3.3 (1.6–6.9)	0.002
13	8/20 (40.0)	2.2 (0.9–5.9)	0.102	4.3 (1.5–12)	0.006
7C	3/27 (11.1)	0.4 (0.1–1.5)	0.178	0.5 (0.1–1.8)	0.293
35B	14/53 (26.4)	1.2 (0.6–2.5)	0.602	1.8 (0.8–3.8)	0.148
23A	4/18 (22.2)	1 (0.3–3.1)	0.950	2 (0.6–6.7)	0.277
6C	2/10 (20.0)	0.8 (0.2–4.2)	0.834	1.6 (0.3–8.6)	0.579
Age group, y					
<1	152/504 (30.2)	3.5 (2.3–5.3)	<0.001	3.1 (1.9–5.1)	<0.001
1–4	60/319 (18.8)	1.9 (1.2–3)	0.009	1.6 (0.9–2.6)	0.086
5–14	30/272 (11.0)	Referent	Referent	Referent	Referent
Sex					
F	124/505 (24.6)	Referent	Referent	Referent	Referent
M	117/588 (20.0)	0.8 (0.6–1)	0.065	0.7 (0.5–1)	0.055
HIV status					
Negative	111/587 (18.9)	Referent	Referent		
Positive	66/318 (20.8)	1.1 (0.8–1.6)	0.504		
Diagnosis†					
Nonmeningitis	129/695 (18.6)	Referent	Referent	Referent	Referent
Meningitis	113/400 (28.3)	1.7 (1.3–2.3)	<0.001	1.8 (1.3–2.6)	<0.001
Year‡					
2012	40/237 (16.9)	Referent	Referent		
2013	45/193 (23.3)	1.5 (0.9–2.4)	0.097		
2014	38/194 (19.6)	1.2 (0.7–2)	0.468		
2015	33/138 (23.9)	1.5 (0.9–2.6)	0.098		
2016	39/136 (28.7)	2 (1.2–3.3)	0.008		
2017	25/106 (23.6)	1.5 (0.9–2.7)	0.145		
2018	22/91 (24.2)	1.6 (0.9–2.8)	0.133		
Province					
Eastern Cape	19/55 (34.6)	4.3 (1.8–10.5)	0.001	3.9 (1.5–10.1)	0.004
Free State	9/83 (10.8)	Referent	Referent	Referent	Referent
Gauteng	97/417 (23.3)	2.5 (1.2–5.2)	0.014	2.3 (1.1–5)	0.031
KwaZulu–Natal	30/180 (16.7)	1.6 (0.7–3.6)	0.220	1.7 (0.7–3.8)	0.243
Limpopo	3/20 (15.0)	1.5 (0.4–5.9)	0.605	1.1 (0.3–4.9)	0.855
Mpumalanga	5/33 (15.2)	1.5 (0.5–4.8)	0.522	1.2 (0.4–4.3)	0.724
Northern Cape	10/36 (27.8)	3.2 (1.2–8.6)	0.025	3.8 (1.3–11.2)	0.014
North West	9/26 (34.6)	4.4 (1.5–12.6)	0.007	3.3 (1.1–10.6)	0.040
Western Cape	60/245 (24.5)	2.7 (1.3–5.6)	0.010	2 (0.9–4.5)	0.085

*Empty fields indicate that the model did not calculate numbers due to small amount of data or it was not included in the final multivariable model. Factors evaluated in the univariable model but not significant included HIV status, race and underlying medical conditions. Serotypes 7F, 9V and 33F did not have any fatal cases and serotype 2 was not detected. †Meningitis cases include patients diagnosed with meningitis and those with concurrent meningitis and lower respiratory tract infection; nonmeningitis cases include all other IPD cases. ‡The risk factor "year" was significant upon univariate but not multivariate analysis and therefore dropped for the final model.

**Table 4:** Multivariable analysis of factors associated with in-hospital death among patients >15 years of age with invasive pneumococcal disease, South Africa, 2012–2018

Risk factor	No. deaths/total no. (%)	Unadjusted OR (95% CI)	p value	Adjusted OR (95% CI)	p value
Serotype					
4	64/208 (30.8)	0.9 (0.6–1.2)	0.406	1.1 (0.7–1.8)	0.541
6B	14/34 (41.2)	1.3 (0.7–2.8)	0.415	1.6 (0.7–1)	0.296
9V	7/31 (22.6)	0.6 (0.2–1.3)	0.194	0.6 (0.2–1.8)	0.344
14	20/54 (37.0)	1.1 (0.6–2.1)	0.681	1.5 (0.8–3)	0.236
18C	13/37 (35.1)	1 (0.5–2.1)	0.907	0.7 (0.3–1.9)	0.524
19F	47/109 (43.1)	1.5 (0.9–2.3)	0.091	1.5 (0.9–2.5)	0.154
23F	31/79 (39.2)	1.2 (0.8–2.1)	0.395	1.6 (0.9–2.9)	0.127
1	87/202 (43.1)	1.5 (1–2.1)	0.038	1.7 (1.1–2.7)	0.017
3	88/251 (35.1)	1 (0.7–1.5)	0.823	1.1 (0.8–1.7)	0.524
5	7/14 (50.0)	1.9 (0.7–5.6)	0.230	2.3 (0.7–7.8)	0.180
6A	42/87 (48.3)	1.8 (1.1–2.9)	0.015	1.6 (0.9–2.8)	0.127
7F	23/79 (29.1)	0.8 (0.5–1.3)	0.388	1 (0.5–1.8)	0.885
19A	97/306 (31.7)	0.9 (0.6–1.2)	0.499	1.3 (0.9–2)	0.176
2	1/2 (50.0)	1.9 (0.1–31.1)	0.644	4.5 (0.3–74.4)	0.288
8	121/354 (34.2)	Referent	Referent	Referent	Referent
9N	34/112 (30.4)	0.8 (0.5–1.3)	0.454	1.3 (0.8–2.3)	0.307
10A	35/84 (41.7)	1.4 (0.8–2.2)	0.199	1.5 (0.8–2.6)	0.185
11A	12/34 (35.3)	1.1 (0.5–2.2)	0.896	0.9 (0.3–2.7)	0.881
12F	97/307 (31.6)	0.9 (0.6–1.2)	0.481	0.8 (0.5–1.2)	0.250
15B/C	38/80 (47.5)	1.7 (1.1–2.8)	0.027	2.1 (1.2–3.7)	0.010
17F	23/87 (26.4)	0.7 (0.4–1.2)	0.169	0.7 (0.4–1.3)	0.250
20	2/13 (15.4)	0.4 (0.1–1.6)	0.177	0.3 (0–2.8)	0.309
22F	39/99 (39.4)	1.3 (0.8–2)	0.338	1.3 (0.7–2.2)	0.413
33F	3/11 (27.3)	0.7 (0.2–2.8)	0.635	0.8 (0.2–3.9)	0.781
16F	52/121 (43.0)	1.5 (1–2.2)	0.083	1.6 (1–2.7)	0.066
15A	42/104 (40.1)	1.3 (0.8–2)	0.246	1.2 (0.6–2)	0.629
13	19/71 (26.8)	0.7 (0.4–1.2)	0.226	0.8 (0.4–1.6)	0.534
7C	20/59 (33.9)	1 (0.6–1.8)	0.966	1.4 (0.7–2.7)	0.342
35B	19/52 (36.5)	1.1 (0.6–2)	0.738	1 (0.5–2.1)	0.922
23A	19/62 (30.7)	0.9 (0.5–1.5)	0.587	0.6 (0.3–1.2)	0.141
6C	11/34 (32.4)	0.9 (0.4–2)	0.830	0.8 (0.3–2)	0.590
Age group, y					
15–24	86/282 (30.5)	Referent	Referent	Referent	Referent
25–44	549/1,738 (31.6)	1.1 (0.8–1.4)	0.714	1.2 (0.8–1.7)	0.392
45–64	385/917 (42.0)	1.6 (1.2–2.2)	0.001	2 (1.4–2.9)	<0.001
>65	107/240 (44.6)	1.8 (1.3–2.6)	0.001	3.1 (1.8–5.2)	<0.001
Sex					
F	557/1,679 (33.2)	Referent	Referent	Referent	Referent
M	568/1,495 (38.0)	1.2 (1.1–1.4)	0.005	1.3 (1.1–1.6)	0.003
HIV status†					
Negative	154/556 (27.7)	Referent	Referent	Referent	Referent
Positive	618/2,007 (30.8)	1.2 (0.9–1.4)	0.160	1.3 (1–1.7)	0.021
Diagnosis‡					
Nonmeningitis	607/2,138 (28.4)	Referent	Referent	Referent	Referent
Meningitis	520/1,039 (50.1)	2.5 (2.2–2.9)	<0.001	2.8 (2.3–3.4)	<0.001
Year					
2012	205/555 (36.9)	1.4 (1.1–1.8)	0.013	1.1 (0.8–1.5)	0.549
2013	144/486 (29.6)	Referent	Referent	Referent	Referent
2014	143/407 (35.1)	1.3 (1–1)	0.08	1.1 (0.8–1.5)	0.626
2015	177/465 (38.1)	1.5 (1.1–1.9)	0.006	1.4 (1.0–2.0)	0.031
2016	168/449 (37.4)	1.4 (1.1–1.9)	0.012	1.3 (0.9–1.8)	0.106
2017	159/447 (35.6)	1.3 (1–1.7)	0.053	1.3 (0.9–1.8)	0.140
2018	131/368 (35.6)	1.3 (1–1.8)	0.065	1.4 (1.0–1.9)	0.082
Province					
Eastern Cape	77/217 (35.5)	0.8 (0.5–1.3)	0.406		
Free State	55/138 (39.9)	Referent	Referent		
Gauteng	410/1,219 (33.6)	0.8 (0.5–1.1)	0.145		
KwaZulu–Natal	176/505 (34.9)	0.8 (0.5–1.2)	0.278		
Limpopo	29/56 (51.8)	1.6 (0.9–3)	0.130		
Mpumalanga	65/139 (46.8)	1.3 (0.8–2.1)	0.246		
Northern Cape	63/178 (35.4)	0.8 (0.5–1.3)	0.416		
North West	42/93 (45.2)	1.2 (0.7–2.1)	0.423		
Western Cape	210/632 (33.2)	0.8 (0.5–1.1)	0.138		

*Underlying medical conditions as a risk factor was analyzed but not significant in the univariate model. Empty fields indicate that the model did not calculate numbers due to small amount of data, or it was not included in the final multivariable model. Factors evaluated in the univariable model but not significant were race and province. †HIV status was not significant upon initial multivariate analysis but was kept in the final analysis because of the high percentage of HIV positive cases in our study population (78%). ‡Meningitis cases include patients diagnosed with meningitis and those simultaneously diagnosed with meningitis and lower respiratory tract infection; nonmeningitis cases include all other cases.

When we restricted analysis to patients >15 years of age we found that, compared with serotype 8, those infected with serotype 15B/C (OR 2.1, 95% CI 1.2–3.7) were more likely to die (Table 4). Other factors significantly associated with in-hospital death were sex (male, OR 1.3, 95% CI 1.1–1.6), increasing age (45–64 years, OR 2.0, 95% CI 1.4–2.9; >65 years, OR 3.1, 95% CI 1.8–5.2; vs. 15–24 years), and meningitis (OR 2.8, 95% CI 2.3–3.4) (Table 4). In the nmIPD multivariable analysis of patients >15 years, no serotypes were significantly associated with death when compared with serotype 8 (Appendix 2 Table 4).

### Multivariable Analysis of In-Hospital Outcome in Pneumococcal Meningitis

Multivariable analysis indicated that meningitis patients infected with serotype 1 were more likely to die (OR 2.3, 95% CI 1.2–4.1) than those infected with serotype 8 (Appendix 2 Table 3). Other factors significantly associated with increased in-hospital death were positive HIV status (OR 3.2, 95% CI 2.2–4.6), underlying medical condition (OR 1.6, 95% CI 1.1–2.1), and age. Compared with patients 5–14 years of age, those at greatest risk were patients <1 year (OR 4.7, 95% CI 2.2–10.3), 1–4 years (OR 4.3, 95% CI 1.9–10.0), and 45–64 years (OR 7.0 95%, CI 3.4–14.4) (Appendix 2 Table 3).

When we restricted meningitis cases to patients <15 or >15 years of age, we did not find any serotypes significantly associated with increased in-hospital death compared with serotype 8 (Figure 5; Appendix 2 Table 5). Other factors significantly associated with increased odds for in-hospital death in patients <15 years of age were province, positive HIV status (OR 3.1, 95% CI 1.5–6.3), and age (<1 year, OR 5.0, 95% CI 1.9–12.9; 1–4 years, OR 5.4, 95% CI 2.0–14.4) (Appendix 2 Table 5). Other factors significantly associated with increased CFR in meningitis patients >15 years were sex (male, OR 1.6, 95% CI 1.2–2.2), positive HIV status (OR 3.5, 95% CI 2.2–5.6), and underlying medical condition (OR 1.7, 95% CI 1.2–2.6) (Appendix 2 Table 5).

## Discussion

Nine years after PCV7 introduction and 5 years after PCV13 introduction in South Africa, the non-PCV13 serotype 8 is the most common serotype found in IPD patients followed by 19A, 12F, and 3. Serotype 1, a PCV13 serotype decreasing in total number over the study period, was associated with higher CFR than serotype 8 in analysis of meningitis cases including all ages. Serotype 6A was associated with higher CFR in the analysis of IPD for all ages, in particular in IPD cases of patients <15 years of age. In patients >15 years of age, we found that serotype 6A decreased in 2012 and 2013 but then stabilized until study end. Serogroup 15 non-PCV13 serotypes 15A and 15B/C were also associated with higher CFR than serotype 8. Other factors that we found were independently associated with death in >2 analyses were HIV status, age, meningitis infection, year, and province.

Serotypes associated with death in the prevaccine era (2003–2008) in South Africa in patients >15 years of age included serotypes 1 and 19F in IPD patients but no particular serotype in meningitis patients (*19*). Serotype 1, which is a PCV13 but not a PCV7 serotype, was associated with death in our analysis of meningitis cases of all ages in the vaccine era. Serotype 1 is associated with outbreaks of meningitis (*20*,*21*). In studies from mainly prevaccine years (1990–2009) (*22*,*23*), and particularly in a meta-analysis including studies from 1928–2010 (*24*), the risk for death from serotype 1 was found to be lower than that for other serotypes. In the prevaccine era, serotype 1 has been associated with a high invasive disease potential (*25*,*26*) (studies from France, Alaska, and Iceland) and noted to be one of the most common IPD serotypes globally in children <5 years of age (*27*). These studies cover different time periods and geographic locations than ours; distinct serotype 1 lineages circulating in South Africa, as described in du Plessis et al. (*28*), could explain the differences we found in association with death. Similar to a previous study of serotype 1 epidemiology in South Africa in 2003–2013 (*29*), we notice a continued decline of overall numbers of serotype 1 IPD cases in patients >15 years of age. The characteristics of serotype 1 pneumococci lineages and our results suggest that even though numbers are decreasing overall, this serotype should be monitored locally and globally in the future and the association of specific lineages with death and with meningitis should be analyzed.

Vaccines continue to play a crucial role in protection against pneumococcal disease. On the basis of our results in South Africa, we recommend protecting against the PCV13 serotypes 3, 6A, 19F, and 19A, as well as serotype 1, and monitoring the non-PCV13 serotype 12F for its effects on pneumococcal disease. Serotype 6A in particular was associated with death in analysis of IPD of all ages and of IPD in patients <15 years of age. Serotype 19F was associated with death in IPD patients in the vaccine era as well as in the prevaccine era (*19*); a component of both the PCV13 and PCV7 vaccines, serotype 19F seems to be associated with more severe disease both in our study death and in previous studies (*14*,*24*,*30*). Serotype 19A was the second most common serotype in IPD in both the prevaccine and the vaccine era; it is included in the PCV13 but not in the PCV7 vaccine and was noted to be one of the most important replacement serotypes in the United States in children <5 years of age (*31*). Serotype 3 (a PCV13 but not PCV7 serotype) and serotype 12F (a non-PCV serotype) were both among the 4 most common serotypes overall in our IPD study population; the total number of deaths was ≈100 for each. Although we did not find either serotype 3 or 12F independently associated with death in our analysis, the total numbers lead us to suggest monitoring these serotypes in the future.

Although serotype 1 was the most common serotype in the prevaccine era in South Africa (*19*), we found serotype 8 to be the most common in the current vaccine era. This finding in South Africa is similar to a recent report from Europe, North America, and Australia (*32*) and also a study summarizing serotypes globally (*33*). Including this serotype in new vaccine formulas could therefore reduce overall IPD cases. Serogroup 15 should be considered in new vaccine formulations, because we found 15B/C (IPD patients >15 years) and 15A (IPD patients <15 years) to be associated with death. To our knowledge, recent data of postvaccine dynamics of serogroup 15, as well as its association with CFR, are scarce. Savinola et al. (*34*) described the emergence of a 15A multidrug-resistant lineage as a result from a 19A capsule switch and van der Linden et al. (*35*) showed an increase in 15A after PCV-13 introduction in Germany. An increase of serogroup 15 in children with pneumococcal disease was noted in Hong Kong; Liyanapathirana et al. suggested consideration in future vaccine strategies (*36*). Because of these recent studies and the association we found with death, we suggest close monitoring of serogroup 15 in both adults and children.

Our study has limitations that make a direct comparison to other studies difficult. We used data from enhanced surveillance sites for available patient data; doing so excluded cases from smaller and rural hospitals and clinics. We also excluded from analysis cases for which serotype and outcome information were not available. Both limitations may have caused selection bias. Another limitation is that our 2 groups are not mutually exclusive; our analysis of IPD cases includes the meningitis cases, which we also analyzed separately as a subset. The inclusion of meningitis cases in the IPD analysis may be causing some of the serotypes to be associated with CFR. Temporal variation of serotypes after introduction of vaccines was not analyzed; although we considered the increase or decrease of serotypes in the conclusions, the consequences of these variations are hard to anticipate and may have introduced bias. In addition, we did not control for treatment or antimicrobial resistance, which may need to be studied in a future analysis. An advantage of this study is that, in an effort to detect differences in age groups, we analyzed the data in all ages as well as in 2 different age groups of <15 years and >15 years. Another advantage is that we conducted the analysis to detect possible differences of serotype associated with death in IPD cases as well as in a subset of meningitis cases.

In conclusion, our data suggest that PCV13 serotypes 1, 3, 6A, 19A, and 19F should continue to be monitored in surveillance studies. Although we cannot exclude that other serotypes may also be important, we recommend inclusion of serotypes 8 and 12F and serogroup 15 (serotypes 15A and 15B/C) in new vaccines, which may contribute to overall reduction of disease caused by *S. pneumoniae.*

Appendix 1 Additional figures for study of *Streptococcus pneumoniae* serotypes associated with death, South Africa, 2012–2018.

Appendix 2 Additional tables for study of *Streptococcus pneumoniae* serotypes associated with death, South Africa, 2012–2018.
