# Supplementary material for: Streptococcus pneumoniae Serotypes Associated with Death, South Africa, 2012–2018
Source: Emerg Infect Dis. 2022 Jan;28(1):166–79. doi: 10.3201/eid2801.210956 (PMC8714227; doi:10.3201/eid2801.210956)
Supplement: Appendix 1 — Additional figures for study of Streptococcus pneumoniae serotypes associated with death, South Africa, 2012–2018. [file 21-0956-Techapp-s1.pdf]

Appendix DOI: https://doi.org/10.3201/eid2801.210956

Streptococcus pneumoniae Serotypes Associated with Death, South Africa, 2012–2018

Appendix

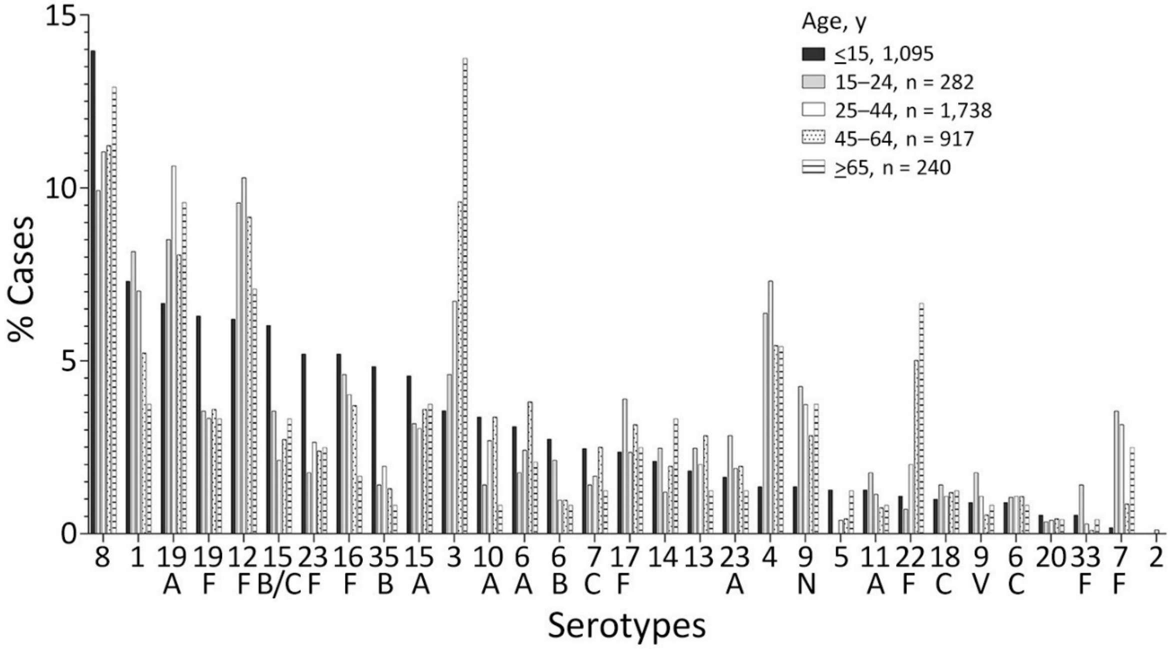

Appendix Figure 1. Distribution of pneumococcal serotypes among invasive pneumococcal disease patients in South Africa in 2012–2018 by age group.

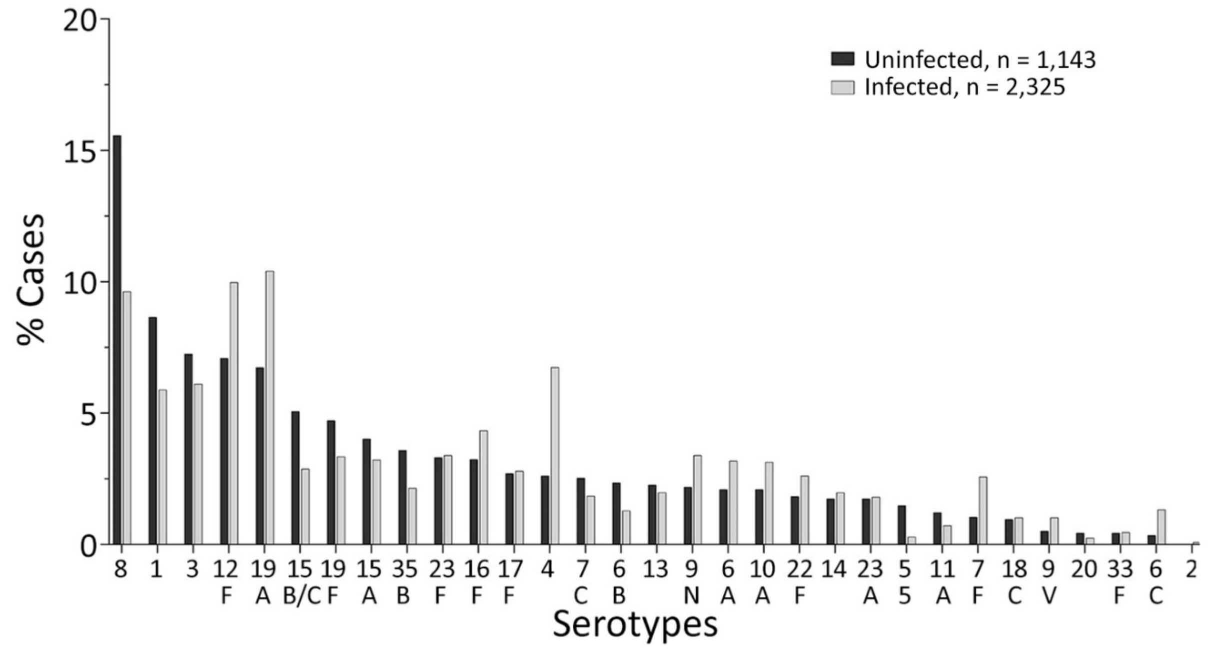

Appendix Figure 2. Distribution of pneumococcal serotypes among invasive pneumococcal disease patients in South Africa in 2012–2018 by HIV infection status.

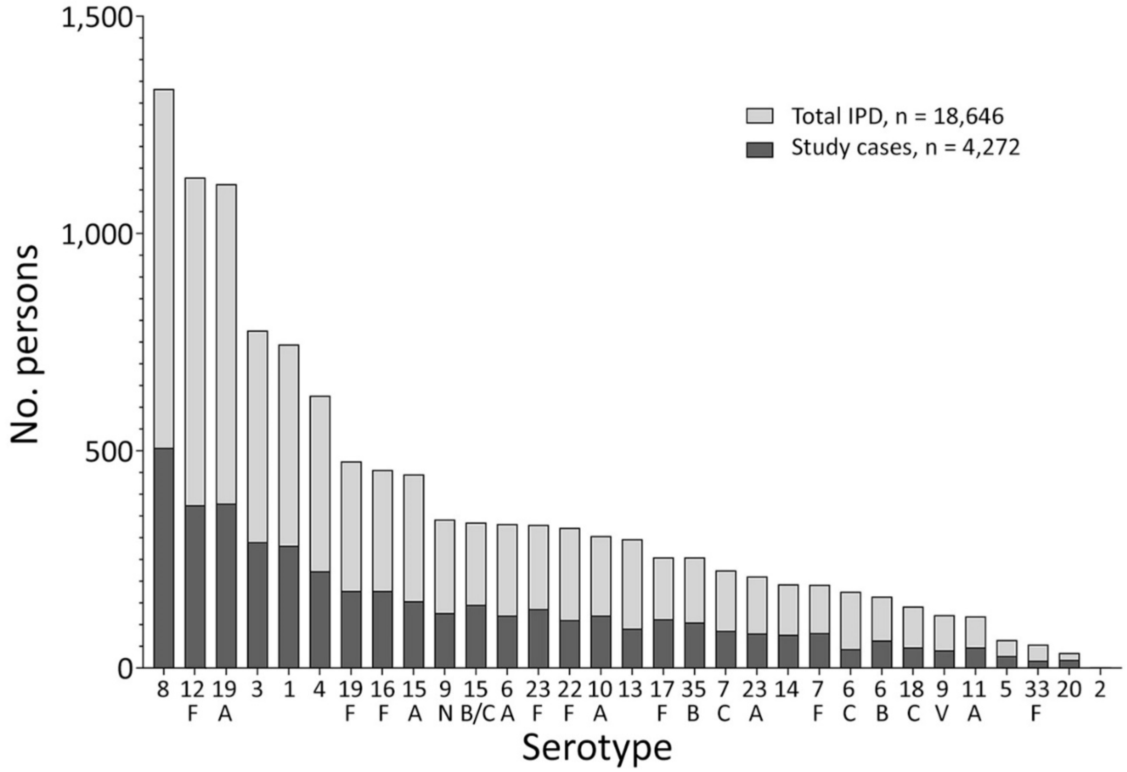

Appendix Figure 3. Isolates per serotype in all invasive pneumococcal disease patients in South Africa in 2012–2018, showing serotype 8 as the most common serotype identified in both total IPD cases and in our study cases. IPD, invasive pneumococcal disease.

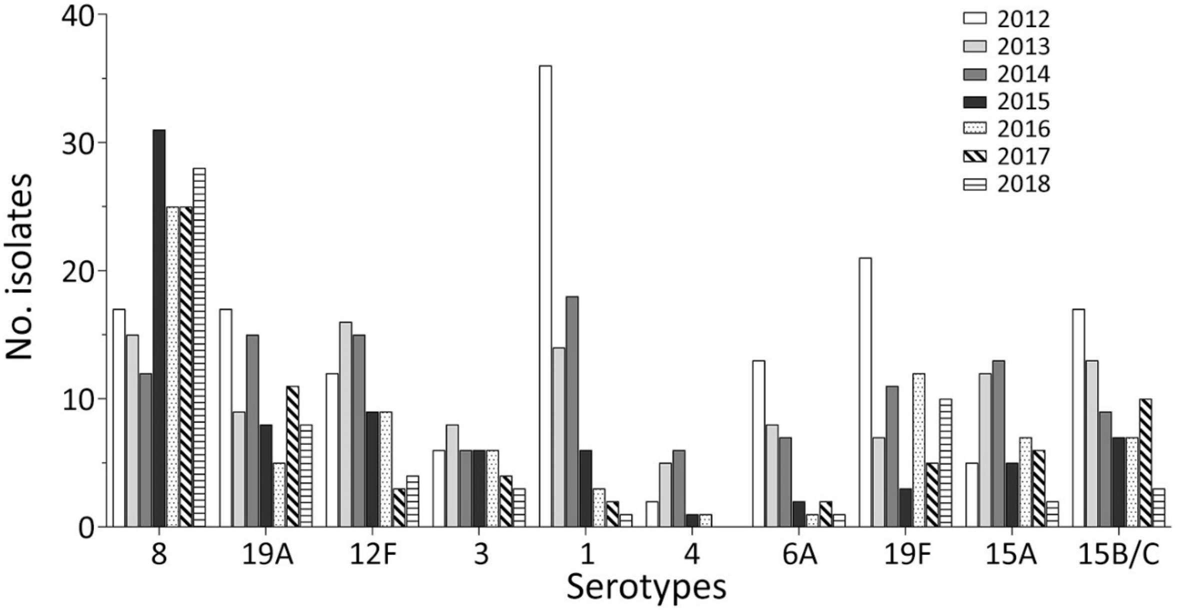

Appendix Figure 4. Isolates per serotype in invasive pneumococcal disease patients in South Africa (2012–2018) in patients <15 years of age of the 4 most common serotypes in the prevaccine era in ages ≥15 years (1, 19A, 3, and 4) as reported in Cohen et al. (*9*), the 4 most common in the vaccine era (8, 19A, 12F, and 3) and 15A, 15B/C, 6A, and 19F. PCV7 was introduced in 2009, PCV13 in 2011.

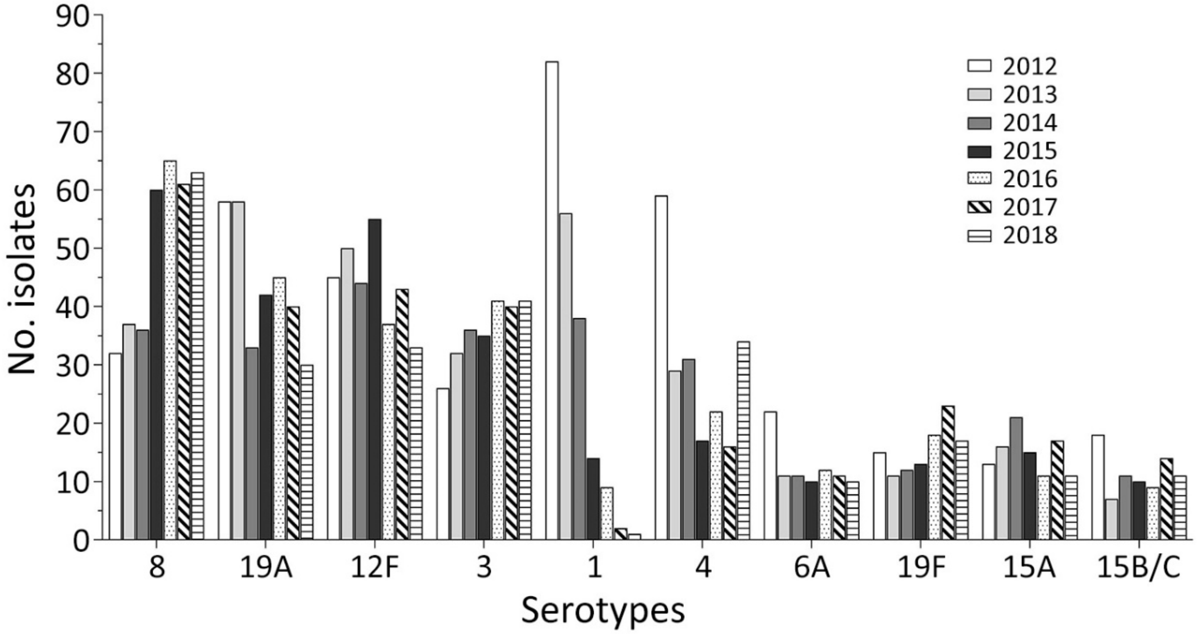

Appendix Figure 5. Isolates per serotype in invasive pneumococcal disease patients in South Africa in 2012–2018 in patients ≥15 years of age of the 4 most common serotypes in the prevaccine era (1, 19A, 3, and 4) as reported in Cohen et al. (*9*), the 4 most common in the vaccine era (8, 19A, 12F, and 3) and 15A, 15B/C, 6A and 19F. PCV7 was introduced in 2009, PCV13 in 2011.

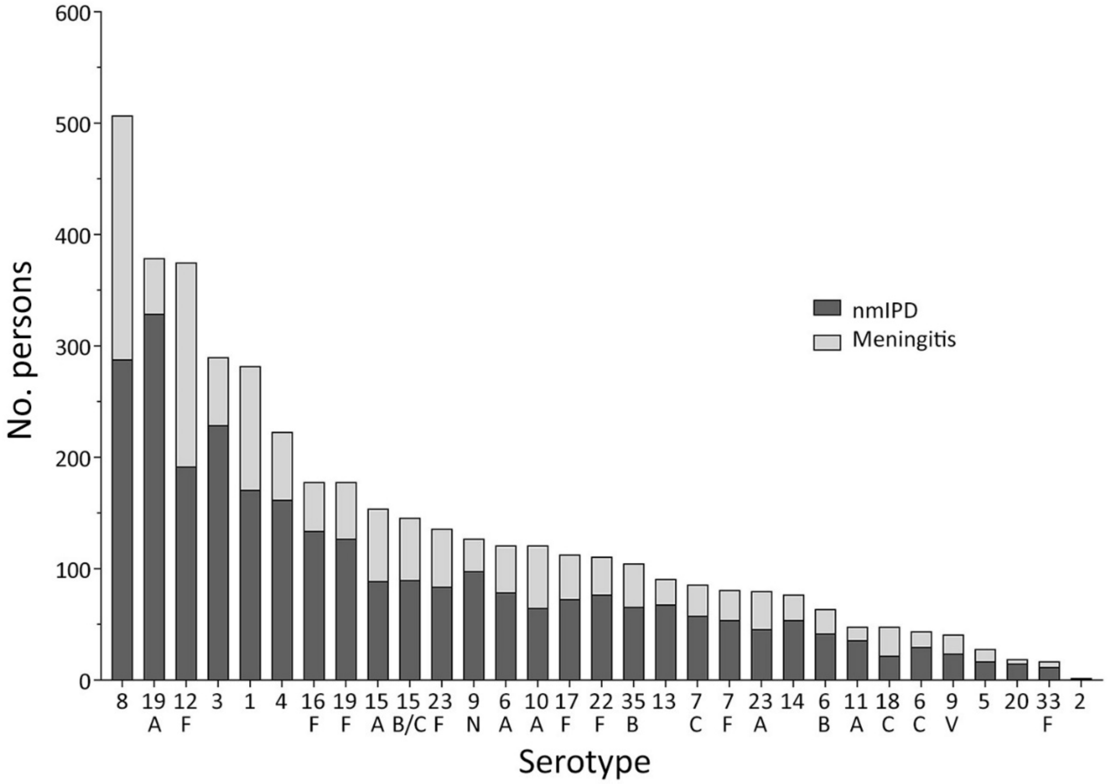

Appendix Figure 6. Isolates per serotype in all invasive pneumococcal disease patients (n = 4,272) depicted as the sum of the number of nonmeningitis cases (dark gray) and meningitis cases (light gray) per serotype in South Africa in 2012–2018. Serotype 8 was the most common overall and in meningitis cases and 19A the most common in nonmeningitis cases. nmIPD, nonmeningitis invasive pneumococcal disease.
